# Engineering an Enhanced EGFR Engager: Humanization of Cetuximab for Improved Developability

**DOI:** 10.3390/antib11010006

**Published:** 2022-01-13

**Authors:** Dennis R. Goulet, Soumili Chatterjee, Wai-Ping Lee, Andrew B. Waight, Yi Zhu, Amanda Nga-Sze Mak

**Affiliations:** 1 Protein Engineering, SystImmune, Inc., 15318 NE 95th St., Redmond, WA 98052, USA; soumilich@gmail.com (S.C.); drewaight@gmail.com (A.B.W.); yi.zhu@systimmune.com (Y.Z.); amanda.mak@systimmune.com (A.N.-S.M.); 2 Process Development, SystImmune, Inc., 15318 NE 95th St., Redmond, WA 98052, USA; waileewpl@gmail.com

**Keywords:** cetuximab, antibody engineering, humanization, glycosylation, post-translational modifications, stability, aggregation, immunogenicity, modeling, binding kinetics

## Abstract

The epidermal growth factor receptor (EGFR) is a receptor tyrosine kinase whose proliferative effects can contribute to the development of many types of solid tumors when overexpressed. For this reason, EGFR inhibitors such as cetuximab can play an important role in treating cancers such as colorectal cancer and head and neck cancer. Cetuximab is a chimeric monoclonal antibody containing mouse variable regions that bind to EGFR and prevent it from signaling. Although cetuximab has been used clinically since 2004 to successfully control solid tumors, advances in protein engineering have created the opportunity to address some of its shortcomings. In particular, the presence of mouse sequences could contribute to immunogenicity in the form of anti-cetuximab antibodies, and an occupied glycosylation site in FR3 can contribute to hypersensitivity reactions and product heterogeneity. Using simple framework graft or sequence-/structure-guided approaches, cetuximab was humanized onto 11 new frameworks. In addition to increasing humanness and removing the VH glycosylation site, dynamic light scattering revealed increases in stability, and bio-layer interferometry confirmed minimal changes in binding affinity, with patterns emerging across the humanization method. This work demonstrates the potential to improve the biophysical and clinical properties of first-generation protein therapeutics and highlights the advantages of computationally guided engineering.

## 1. Introduction

Cetuximab is a chimeric IgG1 monoclonal antibody that was approved in 2004 for the treatment of colorectal cancer and in 2006 for the treatment of squamous cell carcinoma of the head and neck [1,2,3]. By binding to domain III of the extracellular domain of the epidermal growth factor receptor (EGFR), which is overexpressed on tumor cells, cetuximab competitively inhibits the binding of EGF and other ligands, preventing the dimerization of EGFR [1]. The resulting inhibition of receptor tyrosine autophosphorylation leads to reduced EGFR-mediated signaling, which downregulates proliferation, angiogenesis, and metastasis while inducing apoptosis. In addition, the Fc domain of cetuximab binds to CD16a and other Fc receptors in order to recruit immune mechanisms such as antibody-dependent cellular cytotoxicity [4]. In 2020, cetuximab saratolacan, an antibody–dye conjugate that photosensitizes EGFR-bearing tumors, was approved in Japan for the treatment of head and neck cancer, demonstrating the potential of cetuximab binding domains to be used in alternative formats, such as antibody–drug conjugates and multipolicy antibodies [5].

A potential shortcoming of cetuximab is that its variable regions were generated in mice, and those regions retain non-human sequences. It has been demonstrated that chimeric antibodies may have increased capacity for immunogenicity compared to humanized or human antibodies [6]. On the other hand, humanization can increase the stability of antibodies by making the framework regions more compatible [7]. Another concern is the occupied glycan site at VH N85 (Kabat), where Fab glycosylation could affect the biological properties of the antibody as well as introducing glycan heterogeneity, which must be well controlled during manufacturing [8,9]. Although the immunogenicity of cetuximab appears low based on the low incidence of anti-cetuximab IgG responses (5%), hypersensitivity is a common occurrence due largely to pre-existing IgE antibodies against the galactose-α-1,3-galactose oligosaccharide that modifies the VH when expressed in Sp2/0 cells [10,11,12].

To overcome these liabilities, cetuximab was humanized with the goals of removing post-translational modification sites, stabilizing the antibody, and reducing the potential for immunogenicity while retaining a high affinity for EGFR. The strategies used for humanization included a straight CDR graft onto a stable human framework, sequence-guided grafting onto the most similar germline or consensus framework, and a structure-guided approach based on the predicted stability effects of humanizing mutations. The result is a panel of humanized cetuximab sequences with superior biophysical properties, where the structural modeling approach was the most successful in generating stable binders with no loss in EGFR affinity.

## 2. Materials and Methods

### 2.1. Humanization

For humanized version H1, cetuximab Kabat CDRs were grafted onto a stable framework as described previously [13]. All other humanized versions were designed using the Discovery Studio 2020 suite. Versions H2–H5 were designed using the Predict Humanizing Mutations protocol, which is based exclusively on the amino acid sequence of cetuximab as the query sequence. The identity threshold was set to 50, the frequent residue substitution tolerance was set to 20, the germline substitution tolerance was set to 0, and substitutions of Kabat CDR residues, IMGT CDR residues, Vernier zone residues, and human germline residues were excluded. Versions H2 and H3 were generated based on germline substitutions, whereas versions H4 and H5 used frequent residue substitutions. Versions H6–H11 were designed using different input models for cetuximab, with “calculate mutation energy” set to true (CHARMm forcefield) in order to generate the “best single mutations” sequences. The query structure was various models for cetuximab, as shown in Table 1. Version H6 used the cetuximab component of PDB 1YY9 (cetuximab in complex with EGFR) in order to capture the poses of CDRs in the bound state. Versions H7–H11 used the cetuximab models generated by Discovery Studio’s Antibody Modeling Cascade. The input sequences were cetuximab VH and VL for H7, cetuximab VH (ending TVSS instead of TVSA) and VL for H8 and H9, cetuximab VH and VL (ending LTVL instead of LELK) for H10, and cetuximab VH (ending TVSS instead of TVSA) and VL (ending LTVL instead of LELK) for H11. The top five framework templates were used with a sequence similarity cutoff of 10. The CDR loop definition was set to Honegger and the maximum templates per loop was set to 3, with the optimization level set to high. After generating humanized sequences, versions H2, H4, and H8 were modified to H3, H5, and H9, respectively, by substituting the last four residues of the VL to LTVL to mimic the stable FR4 of lambda antibodies.

### 2.2. Sequence Analysis

The humanness of the VH and VL sequences (framework only) was calculated using the Lake Pharma Antibody Analyzer (https://dm.lakepharma.com/bioinformatics/ (accessed on 29 June 2021)), which provides a T20 score (range of 0 to 100, with 100 being the most human) [14].

The MixMHC2pred algorithm (https://github.com/GfellerLab/MixMHC2pred (accessed on 29 June 2021)) was used to predict MHCII-binding ligands within the antibody sequences [15]. The algorithm detects the number of “core” peptides in a given amino acid sequence that will bind to MHCII with sufficient affinity to form a stable T-cell epitope. The higher the number of MHCII-binding peptides identified in a sequence is, the more potential T-cell epitopes the sequence contains. Notably, the algorithm cannot distinguish between immunogenic and tolerogenic peptides; however, a high number of core peptides increases the likelihood of containing some peptides that are pro-immunogenic. The MixMHC2pred algorithm was purchased and downloaded from its GitHub repository. To simplify the scoring system into a single value per VH-VL pair, sequences were run as scFv (VL-(G4S)3-VH), which accounts for peptides within both VH and VL. After running the algorithm on VL/VH scFv sequences containing a (G4S)4 linker, the number of core peptides was calculated and tabulated for the different sequences. Scoring was performed across multiple alleles, allowing sequences to be evaluated for the presence of strong ligands to any allele of MHCII. The number of core peptides was calculated based on the number of peptides in the sequence that could bind to any MHCII allele with a score in the top 0.2% of interactions.

### 2.3. Expression and Purification

The sequences for scFv-monoFc and mAb heavy and light chains were separately cloned into a pTT5 expression vector using the NEBuilder HiFi Assembly (New England Biolabs, E5520S) with custom gene fragments generated by GeneWiz. The sequence for the monoFc domain was from 1-B10-9, as described previously [16]. The transfection of single-expression plasmids (scFv-monoFc) or two-expression plasmids (equal amounts of mAb heavy chain and light chain) was performed in 30 mL of ExpiCHO cells in duplicate for each protein according to the manufacturer’s instructions (Thermo Fisher, A29129, Waltham, MA, USA). After 9 days, the supernatants were harvested by centrifuging the cells at 4000 rpm for 30 min, followed by the filtration of the supernatant through a 0.2 µm filter.

The expression titer was determined by biolayer interometry on an Octet RED384 system using protein A sensors. After measuring the association of scFv-monoFc or mAb proteins in the supernatant to the protein A sensors, the initial slope was used to calculate the titer using the calibration standards generated with previously purified scFv-monoFc or mAb protein.

The purification of scFv-monoFc and mAb proteins from the supernatant was performed using a 1 mL mAb Select PrismA column containing protein A affinity resin (Cytiva, 17549851, Marlborough, MA, USA) on an AKTA Pure FPLC system according to the manufacturer’s instructions. After a 10 mL wash with PBS and elution with 50 mM of sodium acetate at pH 3.6, the eluate was neutralized with 1/5 volume of 1 M sodium acetate, 0.5 M NaCl, at pH 7.0. If necessary, the proteins were further purified by preparative SEC using a Superdex 200 Increase 10/300 column on the AKTA Pure FPLC system to reach >95% pure monomer for further assays. The final buffer composition for all proteins was 25 mM sodium acetate, 75 mM NaCl, 5% (*w*/*v*) sucrose, at pH 5.5.

### 2.4. Analytical Size-Exclusion Chromatography

Immediately after the first-step protein A purification, scFv-monoFc proteins were analyzed by analytical SEC using Waters Acquity UPLC H-Class with the ACQUITY UPLC^®^ Protein BEH SEC 200Å, 4.6mm × 150mm, 1.7 µm column. PBS (125 mM sodium phosphate, 137 mM sodium chloride, pH 6.8) was used as the mobile phase for 10 min runs at 0.3 mL/min, where 10 µg protein was injected. For higher resolution, mAbs were instead analyzed by analytical SEC using an Acquity Arc Waters HPLC with XBridge BEH SEC 300Å, 7.8 × 300 mm, 3.5 µm column. PBS (150 mM sodium phosphate, 100 mM sodium chloride, pH 6.8) was used as a mobile phase for 20 min runs at 0.714 mL/min, where 50 µg protein was injected. Two separate purifications were assessed for each protein, with the % peak of interest values reported as average ± standard deviation.

### 2.5. SDS-PAGE

ScFv-monoFc proteins were analyzed by SDS-PAGE using NuPAGE 4–12% Bis-Tris gels (Thermo Fisher, NP0323BOX) and MES running buffer (Thermo Fisher, NP0002, Waltham, MA, USA). A total of 3 µg of each protein was prepared in LDS sample buffer (Thermo Fisher, NP0007) with or without 10 mM of DTT and heated for 10 min at 70 °C. Gels were run for 50 min at 150 V, stained with SimplyBlue (Thermo Fisher, LC6065, Waltham, MA, USA), and destained with water before imaging.

### 2.6. Cation Exchange Chromatography

Antibodies were analyzed by cation exchange chromatography using Agilent 1260 Infinity Quaternary HPLC with Thermo Scientific ProPac™ SCX-10 HPLC Column, 4 × 250 mm, 10 µm, at 35 °C. Thermo Scientific CX-1 pH Gradient Buffers were used as mobile phases. Buffers A and B have pH values of 5.6 and 10.2, respectively. A total of 50 µg of protein sample was loaded, separated with a flow rate of 0.5 mL/min, and eluted with a gradient over 35 min. The gradient included a 3 min hold at 100% buffer A (0% buffer B), a 2 min ramp to 25% buffer B, a 15 min ramp to 70% buffer B, a 0.01 min ramp to 100% buffer B, a 5 min hold at 100% buffer B, a 0.01 min ramp to 0% buffer B, and a 10 min hold at 0% buffer B. Theoretical pI values were calculated using Expasy ProtParam.

### 2.7. Biolayer Interferometry

The binding kinetics were determined on an Octet RED384 system using anti-human Fc (AHC) sensors (Sartorius, 18-5064). First, a 20 s sensor check (baseline) step was performed and scFv-monoFc or mAb proteins were subsequently loaded for 180 s at 10 µg/mL. After a 60 s baseline step, association with 1:2 serial dilutions (50 nM to 0.78 nM, plus 0 nM reference) of in-house purified His-tagged human EGFR was performed, followed by a 420 s dissociation step. All steps contained protein dilutions in assay buffer, 1× DPBS containing 1% bovine serum albumin, and 0.05% Tween 20 at pH 7.2. Regeneration was achieved using 10 mM of glycine at pH 1.5. The fitting of reference-subtracted data was performed in the Octet Data Analysis 11.1 software, globally fitting all concentrations to a 1:1 binding model. The binding kinetics for each protein were assessed in duplicate, with tabulated values reported as averages ± standard deviations.

### 2.8. Dynamic Light Scattering

Thermal stability was assessed using a Wyatt DynaPro Plate Reader III. Proteins were diluted to 1 mg/mL in 25 mM sodium acetate, 75 mM sodium chloride, 5% (*w*/*v*) sucrose, pH 5.5, at 30 µL/well. The temperature was ramped from 25 °C to 85 °C at 1.0 °C/min while monitoring the radius. Due to difficulties experienced in fitting the differently shaped unfolding curves reproducibly, the temperature at which the radius surpassed 15 nm was used as an objective metric of thermal stability. Samples were run in duplicate, with tabulated values reported as averages ± standard deviations.

### 2.9. T Cell-Dependent Cellular Cytotoxicity

Luciferized BxPC-3 tumor cells (ATCC, CRL-1687) were cultured at 37 °C in 5% CO_2_, in RPMI 1640 media containing 10% fetal bovine serum. A total of 500 tumor cells (20 µL) per well were plated into a 384-well, white, flat-bottom polystyrene TC-treated microplate (Corning 3570) and incubated at 37 °C, 5% CO2. After 24 h, 2500 human pan T cells (20 µL) were added to reach an effector-to-target (E–T) ratio of 5:1, and 10 µL of antibody was added at a 5-fold dilution series to reach a final concentration of 0–30 nM. Cells were dispensed using a Multidrop bulk liquid dispenser (BIOTEK). Plates were incubated for an additional 72 h at 37 °C, 5% CO_2_, before luminescence-based cell viability quantification was performed.

To quantify the luminescence produced by constitutively expressed firefly luciferase, the Bright-Glo Luciferase Assay System (Promega, E2620, Madison, WI, USA) was used. BrightGlo reagent was added (20 µL per well) at room temperature and luminescence was quantified with a luminescence-detecting plate reader (BMG Labtech). Antibody EC50 was determined by transforming the data in Microsoft Excel and analysis was performed with the GraphPad Prism 6 software “log(agonist) vs. response—variable slope (four parameters).” The resulting EC50 value is reported based on quadruplicate measurements.

## 3. Results

### 3.1. Humanization Strategy

To increase the humanness of the cetuximab variable regions and decrease the potential for immunogenicity, the mouse VH and Vκ domains were converted to a more human framework. Version H1 was based on a simple graft of Kabat CDR residues onto a stable human framework termed FW1.4gen [13]. Versions H2 through H5 were designed based on sequence homology to human germline sequences. In particular, for versions H2 and H3, the framework residues were mutated in order to match the most similar human germline sequences. For versions H4 and H5, the framework residues were mutated to the consensus residue in human antibodies. The rest of the humanized versions (H6 through H11) were designed based on a structural analysis of cetuximab by mutating framework residues to those residues occurring with a frequency of at least 5% in the human germline that produced the most stable structure in silico. As the energy analysis for this type of humanization depends on the input model used, several input structures were examined. Version H6 used the cetuximab crystal structure 1YY9 [17]. Versions H7 through H11 used scFv models generated from the antibody modeling feature of Discovery Studio based on the sequence of cetuximab variable domains. Versions 8 through 11 incorporated changes in the input sequence to increase the similarity of the VH C-terminus to the consensus sequence in humans or to make the Vκ C-terminus more Vλ-like. After humanization in Discovery Studio, H3, H5, and H9 were further modified by converting the last three residues of the Vκ domain into their corresponding residues from the λ J-gene. This change was evaluated based on the known importance of the last VL beta strand in determining scFv stability and aggregation propensity, as well as the more hydrophobic nature of the Vλ terminus, which could provide packing energy to stabilize the interaction [13,18,19]. All humanization strategies are summarized in Table 1.

### 3.2. Sequence Analysis

Sequences for cetuximab variable domains and their humanized versions were compared, demonstrating identical sequences in the Kabat CDRs and Vernier zones (flanking the CDRs and in structurally important framework regions). The examination of amino acid identity between the sequences (Figure S1) revealed that the humanized VH sequences had an 84–87% identity with cetuximab and a 79–100% identity with each other. Excluding the comparison of versions with modified lambda J regions, which by definition have a 100% VH identity to their corresponding unmodified humanization, the maximum identity between humanized VH sequences was 95%. The humanized VL sequences had a 79–86% identity with cetuximab and a 76–98% identity with each other. Notably, the sequence identity was reduced when comparing only the framework regions (70–82% identity for cetuximab VH and humanized VH and 60–82% identity for cetuximab VL and humanized VL).

All prominent PTM sites in cetuximab were absent in the humanized sequences. Kabat residue VH N85, comprising an occupied NDT glycosylation motif in cetuximab, was modified to A, D, or E amino acids in the humanizations, eliminating this known glycan site. Similarly, VL N41, comprising an NGS glycosylation motif in cetuximab, was changed to the more typical G residue in all humanizations. The N41G substitution, along with the substation of G42, also eliminates the potential for the deamidation of cetuximab VL N41.

**Figure 1:**
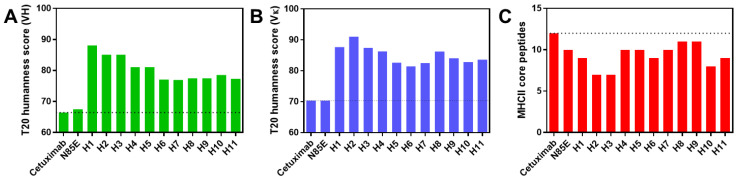
Properties of cetuximab sequences. (**A**) T20 humanness scores for cetuximab-derived VH sequences. (**B**) T20 humanness scores for cetuximab-derived Vκ domains. (**C**) Predicted number of MHCII-binding peptides in cetuximab-derived variable regions based on the MixMHC2pred algorithm. N85E is aglycosylated cetuximab, and H1 through H11 are humanized variants.

The humanness of wild-type cetuximab (mouse variable regions) and humanized variable regions was calculated using the T20 humanness score based on the sequence of the framework regions [14]. Cetuximab, which is a chimeric antibody with mouse variable regions, had low T20 scores of 66.44 (VH) and 70.38 (Vκ). The T20 score of humanized VH domains increased from 66.44 to a range of 76.95–88.10 (Figure 1A), whereas the score of humanized Vκ domains increased from 70.38 to a range of 81.44–91.04 (Figure 1B). Thus, the humanization of cetuximab variable regions significantly improved the humanness of these sequences, which could reduce immunogenicity based on increased sequence homology to human germlines.

Although the presence of non-human sequences in biologics can cause immunogenicity in the form of anti-drug antibodies (ADAs), robust high-affinity ADA can only occur if the offending B cell is activated to undergo class-switch recombination to the IgG subtype. This B-cell activation requires the binding of the presented MHCII-peptide to a compatible T-cell receptor on CD4+ T cells. Thus, an undesired ADA response is more likely to occur if the therapeutic antibody contains peptides that bind stably to MHC class II.

In order to evaluate the presence of MHCII epitopes within cetuximab variable regions, the VH and VL sequences were run through a calculator that predicts MHCII binding affinity. The algorithm, MixMHC2pred, is based on the binding of ~100,000 peptides to different HLA-II alleles [15]. Based on an input sequence, MixMHC2pred evaluates the binding of each peptide within the sequence to each HLA-II allele and returns a ranked score for each residue based on its strongest interaction with any allele. The number of core peptides binding to MHC was calculated based on the number of unique peptides ranking within the top 0.2% of all interactions. To simplify the scoring system into a single value per VH-VL pair, sequences were run as scFv (VL-(G4S)3-VH), which accounts for peptides within both VH and VL. Using this system, the number of MHCII core peptides was calculated for cetuximab and the humanized sequences (Figure 1C). Interestingly, although MHCII binding was not used as a criterion for humanization, all the humanized sequences had a reduced number of peptides scoring within 0.2% of interactions. Whereas cetuximab had 12 core peptides, the humanized sequences had 7–11 core peptides. This reduction in MHCII binding, combined with the more human sequence, may lessen the likelihood of immunogenicity for the humanized variable regions.

### 3.3. Biophysical Properties of Humanized scFv Domains

To assess the biophysical properties of humanized cetuximab VH and VL domains, sequences were cloned into a scFv format and fused to a monoFc to facilitate purification and Octet analysis [16]. The scFv domain was in a VL-VH orientation and included a (G4S)4 linker between the VH and VL domains. This format was selected based on reports that VL-VH orientation could increase retention of activity, and that longer linker length, particularly for VL-VH scFvs, could decrease oligomerization [20,21,22,23]. Notably, the generation of the scFv panel was more efficient than generating the corresponding mAb panel, which requires separate cloning for heavy and light chains. As controls, the wild-type cetuximab scFv and an aglycosylated version of wild-type generated by the mutation of the modified asparagine residue (VH N85E) were also generated.

Plasmids encoding wild-type, aglycosylated (N85E), and humanized scFv-monoFc proteins were transiently transfected in ExpiCHO cells, and protein was purified from the cell supernatant using protein A affinity chromatography. As shown in Table 2, the majority of humanized proteins had a superior expression titer to the wild-type and simple aglycosylated versions of cetuximab, despite containing the same monoFc domain and using the same algorithm for codon optimization. While the average titers for wild-type and aglycosylated cetuximab were 163 and 116 µg/mL, respectively, the average titer for the humanized versions ranged from 220 to 506 µg/mL for H4 and H9, respectively.

**Figure 2:**
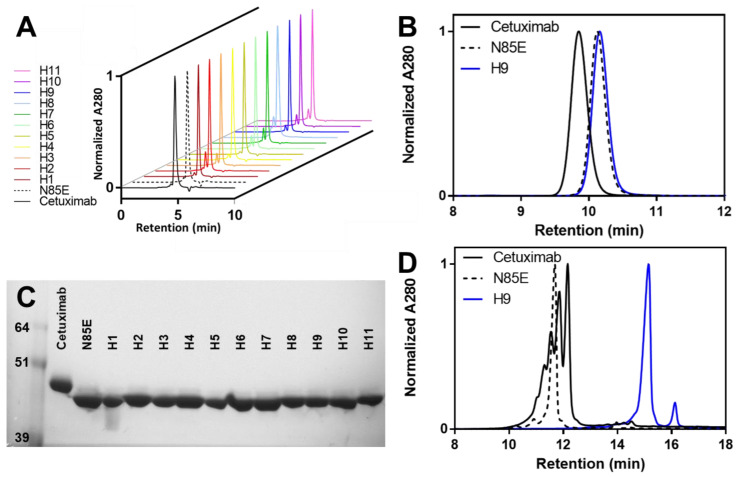
Purification data for cetuximab-derived scFv-monoFc proteins and mAbs. (**A**) Analytical size-exclusion chromatograms for scFv-monoFc proteins immediately after first-step protein A purification demonstrate variable levels of aggregation. (**B**) Analytical size-exclusion chromatography for mAb proteins immediately after first-step protein A purification demonstrates low aggregation and smaller size upon humanization. (**C**) Non-reducing SDS-PAGE of purified scFv-monoFc proteins demonstrates a higher mobility of humanized versions lacking a glycosylation site. (**D**) Cation exchange chromatography of purified mAbs demonstrates an increase in PI and a reduction in charge variants upon humanization. Data are representative of two independent expressions and purifications, except the ion exchange data, which were only collected once. N85E is aglycosylated cetuximab, and H1 through H11 are humanized variants.

After the first-step protein A purification, analytical size-exclusion chromatography (SEC) was used to assess the aggregation of the scFv-monoFc proteins (Table 2, Figure 2A). The wild-type and aglycosylated cetuximab were 93.6% and 94.9% protein of interest, respectively, due to a small amount of aggregation, and the humanized versions had on average similar levels of aggregation. The version with the least aggregation (H5) was 97.4% protein of interest, whereas the most aggregated version (H8) was 82.6% protein of interest. Interestingly, the modification of the Vκ C-terminus to include the sequence from Vλ appeared to decrease aggregation. Humanized versions containing these Vλ residues (H3, H5, and H9) had less aggregation than the corresponding versions where the original Vκ residues were used (H2, H4, and H8, respectively). Preparative SEC was performed for all proteins in order to remove aggregate, exchange into storage buffer, and ensure the accuracy of subsequent biophysical assays, which benefit from using highly pure protein. An SDS-PAGE of purified scFv-monoFc proteins demonstrated the increased mobility of N85E and all humanized versions relative to wild-type cetuximab, confirming the lack of glycosylation for these variants (Figure 2C and S2).

**Figure 3:**
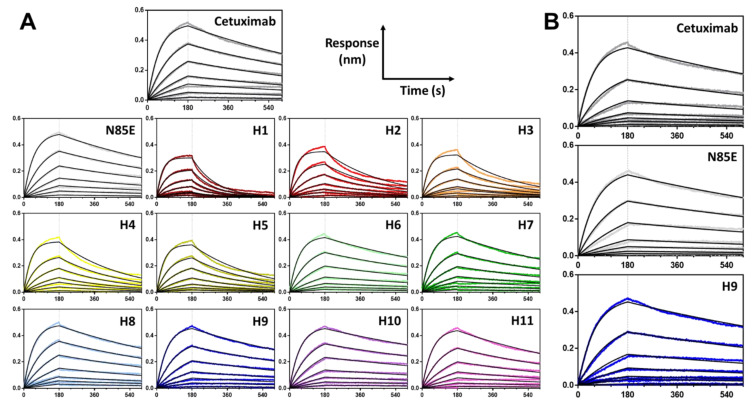
Binding kinetics of cetuximab-derived scFv-monoFc proteins (**A**) and mAbs (**B**), as determined by biolayer interferometry using anti-human Fc (AHC) sensors and soluble recombinant extracellular domain of human EGFR. Data are representative of two independent experiments. K_D_ values are shown in Table 2 and Table 3. N85E is aglycosylated cetuximab, and H1 through H11 are humanized variants.

The binding of scFv-monoFc proteins to human EGFR was assessed by biolayer interferometry to reveal whether the humanization process altered the binding kinetics (Table 2, Figure 3A). The monoFc domain was used to load proteins onto anti-human Fc (AHC) sensors, followed by the binding of scFv to serial dilutions of the extracellular domain of human EGFR. Wild-type cetuximab scFv had an affinity of 3.18 nM, consistent with previous reports. The aglycosylated variant (N85E) had very similar binding kinetics with a K_D_ of 3.16 nM, indicating that glycosylation is not imperative for antigen binding.

The K_D_ values for the humanized versions fell into three main categories. For the humanized version using a straight CDR graft onto a stable human framework (H1), there was a four-fold decrease in binding affinity, which was driven by the increased rate of dissociation. For humanizations based on sequence homology to a single human germline (H2, H3) or the global dataset of human germlines (H4, H5), there was a consistent two-fold decrease in binding affinity, where faster dissociation was again the kinetic determinant. Finally, for humanizations based on structural homology (H6 through H11), there was no significant decrease in binding affinity.

**Figure 4:**
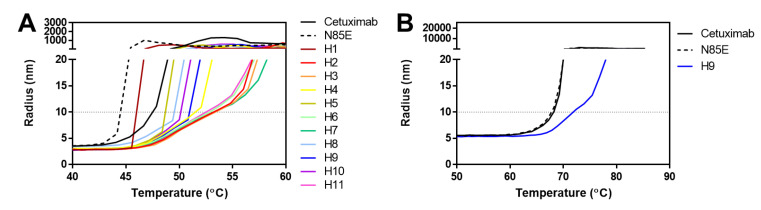
Thermal stability of cetuximab-derived scFv-monoFc proteins (**A**) and mAbs (**B**), as determined by dynamic light scattering. Data are representative of two independent experiments. Unfolding temperatures (the point at which the radius surpassed 10 nm) are shown in Table 2 and Table 3. N85E is aglycosylated cetuximab, whereas H1 through H11 are humanized variants.

Finally, the thermal stability of scFv-monoFc proteins was assessed using dynamic light scattering (Table 2, Figure 4A) by observing the increase in hydrodynamic radius as the temperature was ramped from 25 °C to 85 °C. Since the shapes of the unfolding curves were complex and not uniform for the different samples, the temperature at which the radius surpassed 10 nm was used to objectively compare the protein stabilities. Using this metric, the wild-type cetuximab protein unfolded at 47.2 °C, whereas the aglycosylated N85E variant appeared to be slightly less stable, unfolding at 44.5 °C. Thus, the occupied glycosylation site may help to stabilize the folded conformation of wild-type cetuximab scFv.

Similar to the binding results, three categories of stability were observed. For the humanization based on CDR grafting to an unrelated human framework, the stability was slightly decreased relative to wild-type cetuximab (46.2 °C). Five humanized versions showed a similar or slightly enhanced stability relative to cetuximab (48.7–51.3 °C). Two of these were based on sequence homology to the global dataset of human germlines (H4, H5), whereas three were based on structural modeling (H8, H9, H10). Lastly, five humanized versions appeared to be significantly more stable than the other proteins (unfolding at 52.2–53.0 °C). H2 and H3 were generated by CDR grafting onto the most sequentially homologous human framework, whereas H6, H7, and H11 were based on homology models. In contrast to SEC data showing a systematic reduction in aggregation by using C-terminal residues from λ J genes, the DLS data did not show a consistent impact of these residues on stability. Whereas versions H3 and H9 showed subtle increases in stability relative to H2 and H8, respectively, H5 actually appeared less stable than its relative H4.

### 3.4. Biophysical Properties of Humanized mAb

To understand whether the properties of scFv-monoFc proteins could be translated to an IgG format, mAbs were generated for cetuximab, the aglycosylated variant N85E, and a humanized version of cetuximab. Based on its highest protein expression, low aggregation, improved thermal stability, and unchanged binding affinity, humanized version H9 was selected for conversion to a mAb format. The three mAb proteins were produced by transient transfection in ExpiCHO cells and harvested after nine days of expression.

Mirroring the scFv-monoFc results, the expression titer of humanized H9 was increased relative to that of wild-type or aglycosylated cetuximab (Table 3), although the difference in titer was not as pronounced as that for the scFv-mFc format. After protein A purification, all proteins were found to be >99% pure, as assessed by analytical SEC (Table 3, Figure 2B). Notably, there was significantly less aggregation of the mAbs than the corresponding scFv-mFc proteins, which could be attributed to the intrinsic stability of the IgG backbone relative to scFv and monoFc domains. The SEC data also demonstrated that wild-type cetuximab had a significantly shorter retention time than either the aglycosylated N85E or humanized H9 versions. This difference in apparent molecular size can be attributed to the glycosylation of cetuximab, which is absent from N85E and humanized versions.

To evaluate the differences in properties related to the surface charge, mAbs were analyzed by cation exchange chromatography (Figure 2D). Wild-type cetuximab displayed approximately five peaks of varying intensity, likely related to charge variants caused by variable sialylation. The aglycosylated N85E variant, on the other hand, showed a simpler charge profile with a single main peak. Similarly, the humanized H9 version appeared much more homogeneous than wild-type cetuximab. Thus, the removal of Fab glycosylation via humanization can significantly decrease charge heterogeneity. Based on a shift to the right, humanized cetuximab appeared to be more positively charged than the wild-type version. Consistent with this observation, the sequence-based calculation of the isoelectric point (pI) was 7.92 for cetuximab, 7.66 for the N85E variant, and 8.26 for humanized version H9.

The kinetics of mAbs’ binding to human EGFR were assessed by biolayer interferometry (Table 3, Figure 3B) and demonstrated no difference in binding affinity or kinetics between versions. These results confirmed the results of the scFv-monoFc proteins, which demonstrated that the aglycosylating mutation N85E and the humanization mutations of H9 did not disrupt the interaction of cetuximab CDRs with its antigen. The binding affinity of the mAbs was similar to that of the corresponding scFv-monoFc proteins.

Finally, the DLS experiment was repeated to characterize the stability of the mAbs. Whereas wild-type and aglycosylated cetuximab had very similar stabilities (unfolding at 68.3 and 68.1 °C, respectively), the H9 version had an elevated unfolding temperature of 72.5 °C. Thus, humanized version H9 appears to be more stable than wild-type cetuximab, whether in scFv or mAb format.

### 3.5. T Cell-Mediated Killing by αEGFR × αCD3 Bispecific Antibodies

As a final evaluation of functional activity, bispecific versions of cetuximab were generated and used in a T cell-dependent cellular cytotoxicity (TDCC) assay. The three versions of cetuximab mAb (wild-type, N85E, and H9) contained the K409R mutation in the CH3 domain, which allowed for controlled Fab-arm exchange to occur when incubated with an anti-CD3 antibody containing the complementary F405L mutation. The formation of αEGFR × αCD3-bispecific antibodies from the complementary anti-EGFR and anti-CD3 mAbs was confirmed by cation exchange chromatography (Figure S3).

**Figure 5:**
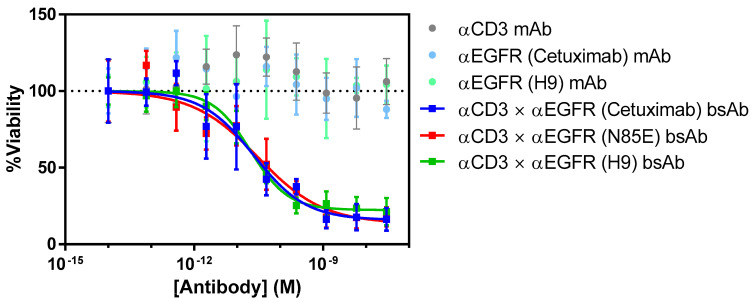
T cell-dependent cellular cytotoxicity (TDCC) assay carried out to assess the functionality of αCD3 × αEGFR bispecific antibodies using wild-type cetuximab, aglycosylated cetuximab (N85E), and humanized version H9 as the αEGFR arm. Luciferized EGFR-bearing BxPC3 target cells were incubated with activated T cells and antibody dilutions for 72 h before reading the luminescence signal representative of viable BxPC3 cells. EC50 values are shown in Table 3.

To assess the TDCC activity, serial dilutions of bispecific antibodies and control mAbs were incubated with activated T cells and luciferized EGFR-bearing BxPC3 target cells at an effector:target ratio of 5:1 in a 384-well plate (Figure 5, Table 3). After three days of incubation at 37 °C, BrightGlo reagent was added to read out the luminescence, which was proportional to the number of remaining target cells. The bispecific cetuximab × αCD3 antibody showed potent tumor cell killing with an EC50 value of 24.6 nM. Agycosylated N85E and humanized H9 showed similar EC50 values (30.7 and 20.3 pM, respectively), with overlapping 95% confidence intervals. In contrast, none of the control mAbs (αCD3, cetuximab, or cetuximab H9) showed any BxPC3 killing up to 30 nM, indicating that cytotoxicity required the simultaneous targeting of both tumor cells and T cells. Thus, the humanized version of cetuximab retained the biological functionality of cetuximab when tested in a TDCC assay.

## 4. Discussion

The humanization of antibodies discovered in non-human species is a common practice not only to decrease their immunogenicity, but also to increase their stability and remove sequence liabilities. In this study, cetuximab scFv was humanized using three distinct strategies, including unbiased CDR grafting, sequence-guided humanization, and model-guided humanization. Although each approach was successful in generating EGFR binders with increased humanness, there was a clear trend in stability and affinity retention across the humanization strategies. Whereas the simple CDR graft resulted in destabilization and the largest (four-fold) loss in antigen affinity, the sequence homology approach resulted in stabilization and a more modest (two-fold) decrease in affinity, and the model-guided approach was the most successful, with significant stabilization and no change in antigen affinity. When expressed as a mAb, the humanized version H9 had an increased titer and thermal stability relative to cetuximab, unchanged binding kinetics, and a similar TDCC potency when transformed to a bispecific αEGFR × αCD3 format. It should be noted that differences in expression host (CHO cells instead of Sp2/0 cells) and formulation (acetate buffer instead of citrate buffer) can affect stability properties, potentially limiting the scope of the observations described here [24].

In addition to their superior biophysical properties, the humanized versions removed the sequence liabilities associated with the mouse variable regions of cetuximab. The humanness of VH and VL was significantly increased in all humanizations, and the presence of immunogenic peptides appeared to be reduced based on the predicted affinity for MHCII alleles. Although it is difficult to predict the immunogenicity of therapeutic antibodies, the increased humanness and decreased number of T cell epitopes could feasibly reduce the incidence of immunogenicity [14,15,25]. Furthermore, the removal of glycosylation and deamidation sites reduces the complexity of lot-to-lot characterization and eliminates the potential for immunogenic Fab saccharides, even when expressed in non-human cells [12,26]. For example, the increase in homogeneity observed by ion exchange indicates that analyses related to surface charge can be greatly simplified for humanized variants that lack glycosylation.

After humanization, the additional modification of the three C-terminal residues of VL was attempted as a means to decrease the aggregation of scFvs [13,18,19]. Due to the absence of CH1/CL domains, scFvs have surfaces that are unnaturally exposed to the solvent. As human Vλ has a more hydrophobic C-terminus with fewer charged residues than Vκ (LTVL vs. LEIK), it may have a superior packing of its last beta sheet. Indeed, this modification decreased aggregation in all three cases (on average by 8%) and increased the titer and thermal stability in two out of three cases.

Cetuximab has been humanized previously using other strategies. One CDR grafting study of cetuximab generated an antibody that could bind to cells overexpressing EGFR, though the affinity was decreased nine-fold [27]. This increase in K_D_ mirrors the affinity change observed for CDR grafting in the present study. Another report demonstrated the successful humanization of cetuximab with no significant affinity change using an in silico approach that predicts the effect of substitutions on humanness and stability [28]. Although the details of energy evaluation differ (e.g., AMBER forcefield rather than CHARMm), this study highlights the power of computational humanization approaches and corroborates the trends observed here. Finally, cetuximab has also been glycoengineered to remove α-1,3-galactose epitopes, demonstrating an alternate approach to decreasing the immunogenicity of this antibody [29].

Collectively, the data presented here demonstrate that the protein engineering of cetuximab can improve its stability and immunogenicity properties, and more generally suggest that sequence- and especially structure-guided methods can be used to generate humanized antibodies with superior stability and binding properties.

## 5. Patents

A US patent application pertaining to this subject matter has been filed by SystImmune, Inc.

## Figures and Tables

**Table 1:** Methods for generating humanized cetuximab variants.

Humanized Version	Method	Basis	Starting Sequence/Model	Modification
H1	CDR graft onto stable framework	Sequence	Cetuximab, FW1.4gen	None
H2	DS germline substitutions	Sequence	Cetuximab	None
H3	DS germline substitutions	Sequence	Cetuximab	Lambda J
H4	DS frequent residue substitutions	Sequence	Cetuximab	None
H5	DS frequent residue substitutions	Sequence	Cetuximab	Lambda J
H6	DS best single mutations	Model	PDB 1YY9	None
H7	DS best single mutations	Model	Cetuximab model	None
H8	DS best single mutations	Model	Cetuximab model (TVSS)	None
H9	DS best single mutations	Model	Cetuximab model (TVSS)	Lambda J
H10	DS best single mutations	Model	Cetuximab model (LTVL)	None
H11	DS best single mutations	Model	Cetuximab model (TVSS-LTVL)	None

**Table 2:** Biophysical properties of cetuximab-derived scFv-monoFc proteins.

Version	Titer (µg/mL)	aSEC %POI	DLS Temp (°C)	EGFR K_D_ (nM)	k_a_ [1/(M·s)] × 10^5^	k_d_ (1/s) × 10^−3^
Cetuximab	163 ± 39	93.6 ± 2.6	47.2 ± 1.0	3.18 ± 0.18	4.44 ± 1.13	1.42 ± 0.44
N85E	116 ± 6	94.9 ± 3.1	44.5 ± 1.3	3.16 ± 0.35	4.44 ± 0.91	1.42 ± 0.44
H1	309 ± 26	91.5 ± 7.1	46.2 ± 0.9	13.1 ± 1.2	7.18 ± 1.68	9.27 ± 1.34
H2	308 ± 3	83.7 ± 1.9	52.6 ± 1.3	6.77 ± 2.10	6.65 ± 0.73	4.58 ± 1.89
H3	376 ± 12	88.6 ± 2.4	53.0 ± 1.3	6.74 ± 1.61	6.61 ± 1.11	4.54 ± 1.81
H4	220 ± 3	92.2 ± 0.0	51.3 ± 1.1	6.79 ± 1.35	5.71 ± 0.78	3.93 ± 1.30
H5	211 ± 22	97.4 ± 0.9	48.7 ± 1.3	6.92 ± 1.58	5.63 ± 0.67	3.95 ± 1.35
H6	411 ± 59	86.8 ± 2.1	52.4 ± 2.1	3.08 ± 0.68	4.81 ± 0.91	1.51 ± 0.61
H7	418 ± 8	88.1 ± 2.7	52.3 ± 2.0	3.45 ± 0.81	5.04 ± 0.92	1.77 ± 0.73
H8	412 ± 5	82.6 ± 5.5	49.7 ± 1.4	3.05 ± 0.62	4.50 ± 0.54	1.39 ± 0.44
H9	506 ± 14	92.7 ± 2.3	50.5 ± 2.4	3.14 ± 0.53	4.47 ± 0.97	1.43 ± 0.54
H10	394 ± 23	85.6 ± 6.2	50.2 ± 1.5	2.27 ± 0.38	4.74 ± 1.31	1.10 ± 0.48
H11	372 ± 73	88.0 ± 3.4	52.2 ± 2.0	3.42 ± 0.36	4.76 ± 1.33	1.65 ± 0.62

Values are the average and standard deviation of two independent experiments.

**Table 3:** Biophysical properties of cetuximab-derived monoclonal antibodies.

Version	Titer (µg/mL)	aSEC %POI	DLS Temp (°C)	EGFR K_D_ (nM)	k_a_ [1/(M·s)] × 10^5^	k_d_ (1/s) × 10^−4^	* EC50 (pM)
Cetuximab	203 ± 13	99.9 ± 0.2	68.3 ± 0.1	2.68 ± 0.07	3.46 ± 0.27	9.26 ± 0.48	24.6 [10.4–58.4]
N85E	209 ± 9	99.4 ± 0.3	68.1 ± 0.9	2.46 ± 0.28	3.26 ± 0.41	7.96 ± 0.09	30.7 [11.3–83.8]
H9	249 ± 1	99.1 ± 0.4	72.5 ± 1.2	2.41 ± 0.15	3.37 ± 0.20	8.12 ± 0.05	20.3 [13.9–29.9]

Values are the average and standard deviation of two independent experiments. * EC50 value for depleting EGFR + BxPC3 cells using αCD3 × αEGFR bispecific antibody, with 95% confidence interval shown in brackets.

## Supplementary Materials

The following supporting information can be downloaded at: https://www.mdpi.com/article/10.3390/antib11010006/s1: Figure S1: Sequence identity matrices; Figure S2: SDS-PAGE of scFv-monoFc proteins; Figure S3: Cation exchange demonstrating the formation of bispecific antibodies.
