# Supplementary material for: Engineering an Enhanced EGFR Engager: Humanization of Cetuximab for Improved Developability
Source: Antibodies (Basel). 2022 Jan 13;11(1):6. doi: 10.3390/antib11010006 (PMC8788544; doi:10.3390/antib11010006)
Supplement: Supplementary file 1 [file antibodies-11-00006-s001.zip › antibodies-1502278-supplementary.pdf]

Engineering an enhanced EGFR engager:

Humanization of cetuximab for improved developability

Dennis R. Goulet, Soumili Chatterjee, Wai-Ping Lee, Andrew B. Waight, Yi Zhu, Amanda Nga-Sze Mak

Contents:

Figure S1: Sequence identity matrices

Figure S2: SDS-PAGE of scFv-monoFc proteins

Figure S3: Cation exchange demonstrating formation of bispecific antibodies

VH identity matrix -- all residues													
	Cetuximab	N85E	H1	H2	H3	H4	H5	H6	H7	H8	H9	H10	H11
Cetuximab		99.2	78.2	84.9	84.9	85.7	85.7	86.6	84.9	84.9	84.9	84.9	84.0
N85E	99.2		79.0	84.9	84.9	86.6	86.6	87.4	85.7	84.9	84.9	85.7	84.9
H1	78.2	79.0		84.9	84.9	92.4	92.4	82.4	82.4	79.8	79.8	81.5	79.8
H2	84.9	84.9	84.9		100.0	92.4	92.4	87.4	85.7	86.6	86.6	89.1	86.6
H3	84.9	84.9	84.9	100.0		92.4	92.4	87.4	85.7	86.6	86.6	89.1	86.6
H4	85.7	86.6	92.4	92.4	92.4		100.0	89.9	89.9	87.4	87.4	89.1	87.4
H5	85.7	86.6	92.4	92.4	92.4	100.0		89.9	89.9	87.4	87.4	89.1	87.4
H6	86.6	87.4	82.4	87.4	87.4	89.9	89.9		95.0	96.6	96.6	95.0	94.1
H7	84.9	85.7	82.4	85.7	85.7	89.9	89.9	95.0		95.0	95.0	94.1	94.1
H8	84.9	84.9	79.8	86.6	86.6	87.4	87.4	96.6	95.0		100.0	93.3	95.8
H9	84.9	84.9	79.8	86.6	86.6	87.4	87.4	96.6	95.0	100.0		93.3	95.8
H10	84.9	85.7	81.5	89.1	89.1	89.1	89.1	95.0	94.1	93.3	93.3		94.1
H11	84.0	84.9	79.8	86.6	86.6	87.4	87.4	94.1	94.1	95.8	95.8	94.1	

VH identity matrix -- framework residues													
	Cetuximab	N85E	H1	H2	H3	H4	H5	H6	H7	H8	H9	H10	H11
Cetuximab		98.9	70.1	79.3	79.3	80.5	80.5	81.6	79.3	79.3	79.3	79.3	78.2
N85E	98.9		71.3	79.3	79.3	81.6	81.6	82.8	80.5	79.3	79.3	80.5	79.3
H1	70.1	71.3		79.3	79.3	89.7	89.7	75.9	75.9	72.4	72.4	74.7	72.4
H2	79.3	79.3	79.3		100.0	89.7	89.7	82.8	80.5	81.6	81.6	85.1	81.6
H3	79.3	79.3	79.3	100.0		89.7	89.7	82.8	80.5	81.6	81.6	85.1	81.6
H4	80.5	81.6	89.7	89.7	89.7		100.0	86.2	86.2	82.8	82.8	85.1	82.8
H5	80.5	81.6	89.7	89.7	89.7	100.0		86.2	86.2	82.8	82.8	85.1	82.8
H6	81.6	82.8	75.9	82.8	82.8	86.2	86.2		93.1	95.4	95.4	93.1	92.0
H7	79.3	80.5	75.9	80.5	80.5	86.2	86.2	93.1		93.1	93.1	92.0	92.0
H8	79.3	79.3	72.4	81.6	81.6	82.8	82.8	95.4	93.1		100.0	90.8	94.3
H9	79.3	79.3	72.4	81.6	81.6	82.8	82.8	95.4	93.1	100.0		90.8	94.3
H10	79.3	80.5	74.7	85.1	85.1	85.1	85.1	93.1	92.0	90.8	90.8		92.0
H11	78.2	79.3	72.4	81.6	81.6	82.8	82.8	92.0	92.0	94.3	94.3	92.0	

VL identity matrix -- all residues													
	Cetuximab	N85E	H1	H2	H3	H4	H5	H6	H7	H8	H9	H10	H11
Cetuximab		100.0	70.1	83.2	81.3	85.0	83.2	86.0	85.0	82.2	79.4	82.2	84.1
N85E	100.0		70.1	83.2	81.3	85.0	83.2	86.0	85.0	82.2	79.4	82.2	84.1
H1	70.1	70.1		79.4	82.2	80.4	83.2	77.6	80.4	80.4	83.2	76.6	78.5
H2	83.2	83.2	79.4		97.2	95.3	92.5	88.8	87.9	91.6	89.7	88.8	88.8
H3	81.3	81.3	82.2	97.2		92.5	95.3	86.9	86.0	89.7	92.5	87.9	86.9
H4	85.0	85.0	80.4	95.3	92.5		97.2	87.9	88.8	89.7	87.9	86.9	88.8
H5	83.2	83.2	83.2	92.5	95.3	97.2		86.0	86.9	87.9	90.7	86.0	86.9
H6	86.0	86.0	77.6	88.8	86.9	87.9	86.0		95.3	95.3	92.5	90.7	90.7
H7	85.0	85.0	80.4	87.9	86.0	88.8	86.9	95.3		95.3	92.5	88.8	91.6
H8	82.2	82.2	80.4	91.6	89.7	89.7	87.9	95.3	95.3		97.2	93.5	90.7
H9	79.4	79.4	83.2	89.7	92.5	87.9	90.7	92.5	92.5	97.2		93.5	89.7
H10	82.2	82.2	76.6	88.8	87.9	86.9	86.0	90.7	88.8	93.5	93.5		91.6
H11	84.1	84.1	78.5	88.8	86.9	88.8	86.9	90.7	91.6	90.7	89.7	91.6	

VL identity matrix -- framework residues													
	Cetuximab	N85E	H1	H2	H3	H4	H5	H6	H7	H8	H9	H10	H11
Cetuximab		100.0	60.0	77.5	75.0	80.0	77.5	81.3	80.0	76.3	72.5	76.3	78.8
N85E	100.0		60.0	77.5	75.0	80.0	77.5	81.3	80.0	76.3	72.5	76.3	78.8
H1	60.0	60.0		72.5	76.3	73.8	77.5	70.0	73.8	73.8	77.5	68.8	71.3
H2	77.5	77.5	72.5		96.3	93.8	90.0	85.0	83.8	88.8	86.3	85.0	85.0
H3	75.0	75.0	76.3	96.3		90.0	93.8	82.5	81.3	86.3	90.0	83.8	82.5
H4	80.0	80.0	73.8	93.8	90.0		96.3	83.8	85.0	86.3	83.8	82.5	85.0
H5	77.5	77.5	77.5	90.0	93.8	96.3		81.3	82.5	83.8	87.5	81.3	82.5
H6	81.3	81.3	70.0	85.0	82.5	83.8	81.3		93.8	93.8	90.0	87.5	87.5
H7	80.0	80.0	73.8	83.8	81.3	85.0	82.5	93.8		93.8	90.0	85.0	88.8
H8	76.3	76.3	73.8	88.8	86.3	86.3	83.8	93.8	93.8		96.3	91.3	87.5
H9	72.5	72.5	77.5	86.3	90.0	83.8	87.5	90.0	90.0	96.3		91.3	86.3
H10	76.3	76.3	68.8	85.0	83.8	82.5	81.3	87.5	85.0	91.3	91.3		88.8
H11	78.8	78.8	71.3	85.0	82.5	85.0	82.5	87.5	88.8	87.5	86.3	88.8	

Figure S1: Sequence identity matrices for cetuximab, aglycosylated cetuximab (N85E), and humanized cetuximab versions (H1-H11). The top panel shows the identity matrix of the entire VH domain, the second panel shows the identity matrix of VH framework regions (entire VH domain, except Kabat CDR residues), the third panel shows the identity matrix of the entire VL domain, and the bottom panel shows the identity matrix of the VL framework regions (entire VL domain, except Kabat CDR residues). Shading from green (maximum 100% identity) to red (minimum 60% identity) is used to facilitate comparison.

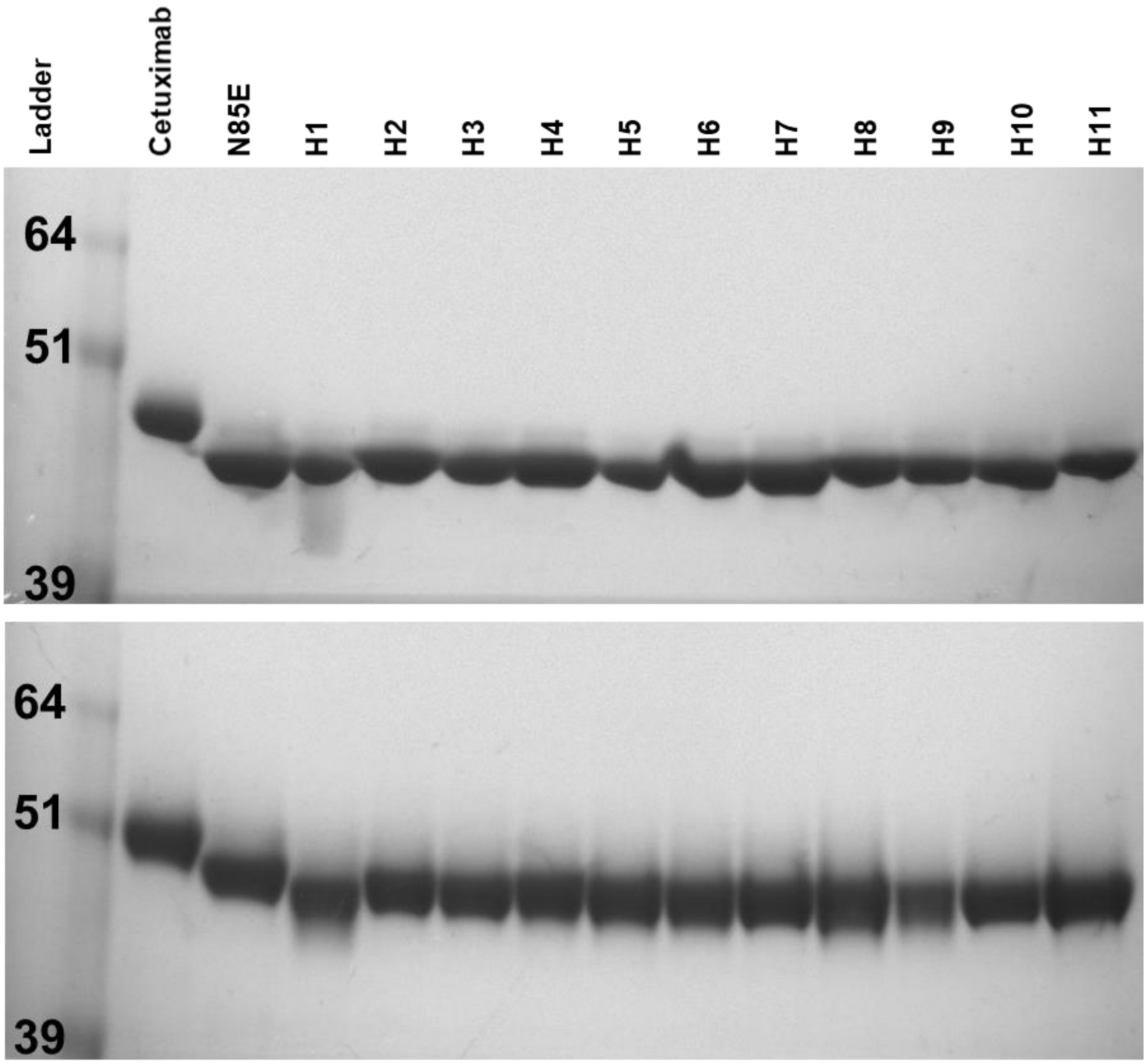

Figure S2: SDS-PAGE of purified scFv-monoFc proteins. Top panel shows non-reducing gel, and bottom panel shows reducing gel with 10 mM DTT.

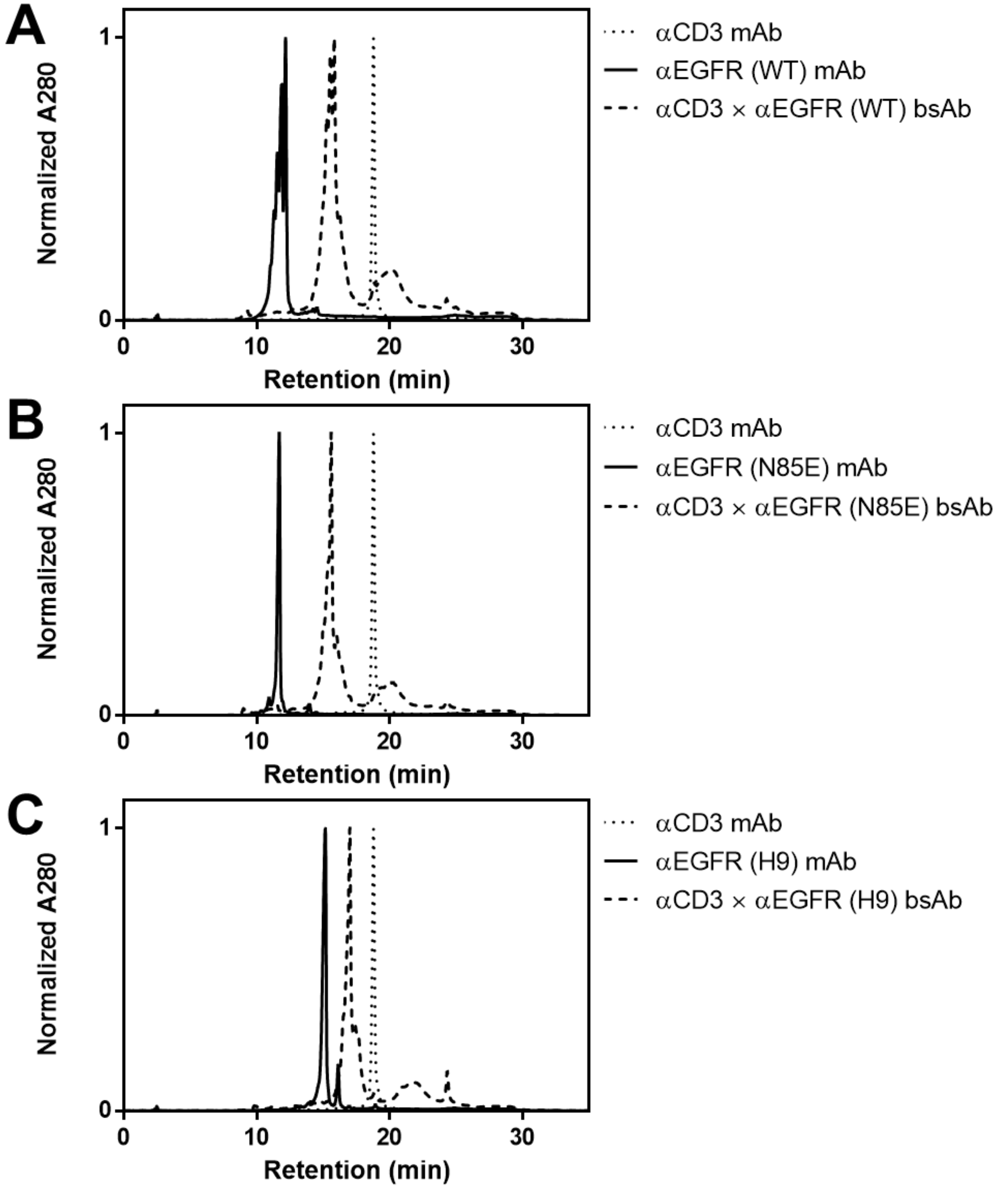

Figure S3: Cation exchange chromatography of parental αCD3 and αEGFR antibodies, as well as resulting αCD3 × αEGFR bispecific antibodies, was used to demonstrate successful formation of bispecific antibodies. Comparison of αCD3 mAb, αEGFR mAb, and αCD3 × αEGFR bispecific antibody is shown for wild-type cetuximab (A), aglycosylated (N85E) cetuximab (B), and humanized (H9) cetuximab (C).
